# Supplementary material for: Non-Invasive Delivery of Negatively Charged Nanobodies by Anodal Iontophoresis: When Electroosmosis Dominates Electromigration
Source: Pharmaceutics. 2024 Apr 13;16(4):539. doi: 10.3390/pharmaceutics16040539 (PMC11055110; doi:10.3390/pharmaceutics16040539)
Supplement: Supplementary file 1 [file pharmaceutics-16-00539-s001.zip › pharmaceutics-2901596-supplementary.pdf]

Supplementary Materials

Non-Invasive Delivery of Negatively Charged Nanobodies by Anodal Iontophoresis: When Electroosmosis Dominates Electromigration

Phedra Firdaws Sahraoui[1,2], *Oscar Vadas*[3], *Yogeshvar N. Kalia*[1,2]

[1] School of Pharmaceutical Sciences, University of Geneva, CMU-1 rue Michel Servet, 1211 Geneva, Switzerland

[2] Institute of Pharmaceutical Sciences of Western Switzerland, University of Geneva, CMU-1 rue Michel Servet, 1211 Geneva, Switzerland

[3] Department of Microbiology and Molecular Medicine, Faculty of Medicine, University of Geneva, Geneva, Switzerland

Supplementary Materials

Phedra Firdaws SAHRAOUI

S1. Competent cells

TOP10 and NovaBlue competent cells were used as cloning strains while, BL21(DE3)$_{WT}$ (wild-type), was used as expression strains.

S2. Site-directed mutagenesis

S2.1. Primer design

The primers used for SDM to generate the mono-substituted variants are listed in the **Table S1** below:

Table S1: *Primers and their respective sequences used for site-directed mutagenesis.*

Primer	*Sequence
Fw R54E	5'GCATCAGCTGGGAGGGTGACAGCACCGGTTATGCG
Rev R54E	5'CGGTGCTGTCACCCTCCCAGCTGATGCCGCTAACAAATTC
Fw K65E	5'CGGATAGCGTGGAGGGCCGTTTCACCATTAG
Rev K65E	5'GAAACGGCCCTCCACGCTATCCGCATAACC
Fw S102E	5'CGGCGGCGGGTGAGGCGTGGTATGGCACCCTGTAC
Rev S102E	5'GCCATACCACGCCTCACCCGCCGCCGCCGCGCAATAG

*Annotations: Codon encoded for the modified amino acid (glutamic acid (E))

S2.2. Polymerase chain reaction

Polymerase chain reaction (PCR) was used to amplify the mutated plasmid DNA used for this project in order to study changes in protein delivery that may occur as a result of these mutations. Q5 High-Fidelity DNA Polymerase was used for amplification from plasmids taken as templates. PCR reaction tubes were prepared as described in **Table S2**.

Table S2: *PCR reaction setup*

Component	20 µl reaction	Final concentration
Q5 High-Fidelity 2X Master Mix	10 µl	1X
Template DNA	variable	< 1.000 ng
Forward Primer (10µM)	1 µl	0.5 µM
Reverse Primer (10µM)	1 µl	0.5 µM
ddH_2O to 20 µl	To 20 µl	

The PCR program used is described in **Table S3.** A temperature gradient was performed (starting from 60°C to 72°C with an increment of 2°C) in order to find the optimal annealing temperature (Ta) for each primer pair (Biometra GmbH, Germany) (**Table S4**).

***Table S3:** Thermocycling Conditions for a Routine PCR:*

Segment	Cycles	Temperature (°C)	Time (min)
1_Initial Denaturation	1	95	1
2_Cycles	18	95<br>60-72 (+2.0)<br>72 (30 sec/kb)	1<br>1<br>5
3_Final Extension	1	72	10
Hold		4-10	

The optimal annealing temperatures (Ta) for each primer pair are listed in the table below:

***Table S4:** Optimal annealing temperatures for each primer pair*

Primer pairs	Ta (°C)
Fw R54E + Rev R54E	68.2
Fw K65E + Rev K65E	68.2
Fw S102E + Rev S102E	71.7

After completion of the PCR, the reaction tubes were placed on ice for 2 minutes to cool the reaction. Afterward, 1 µl (10 U/µl) of *DpnI* restriction enzyme was added directly to each amplification tube in order to digest the methylated parental DNA template (non-mutated plasmid template). The tubes were then incubated at 37°C for 1 hour and the amplified materials were directly transformed into chemically-competent *E.coli* strains (NovaBlue or TOP10).

S3. Heat shock transformation

Plasmids were transformed into competent *E.coli* strains following the procedure below (**Table S5**):

***Table S5:** Heat shock transformation protocol for cloning and expression strains*

Cloning strains	Expression strains
1. Place 50 µl of the competent cells on ice for 5 min	1. Place 10 µl of the competent cells on ice for 5 min
2. Add about 1-10 µl of plasmid	2. Add about 1-10 ng of plasmid

3. Leave on ice for 30 min	3. Leave on ice for 15 min
4. Heat shock at 42°C for 30 seconds	4. Heat-shock at 42°C for 20 seconds
5. Replace on ice for 2-5 min	5. Replace on ice for 2-5 min
6. Add 200-300 µl of LB or SOC broth	6. Add 200-300 µl of LB or SOC broth
7. Incubate for 1 h at 37°C with shaking	7. Incubate for 1 h at 37°C with shaking
8. Plate on pre-warmed selective plates (containing the appropriate antibiotic)	8. Plate on pre-warmed selective plates (containing the appropriate antibiotic)
9. Incubate overnight at 37°C	9. Incubate overnight at 37°C

After transformation, colonies were picked and inoculated into 10 ml LB containing the appropriate selective agent. Plasmids were then re-isolated with the NucleoSpin®plasmid kit according to the manufacturer's instructions and the sequences were verified by sequencing (Microsynth AG, Switzerland). This last step should always be performed for sequence verification and is particularly important in the case of SDM to ensure the success of the reaction. In this latter case, the mutation site was sequenced in both directions, as well as the entire plasmid in order to ensure that no other mutations, except the desired one, occurred in the plasmid. Double-stranded DNA (plasmid) was quantified by the NanoDrop® One^{C} (Thermo Scientific, USA) and glycerol stocks were subsequently prepared, after transformation into expression strains, for further use.

S4. SDS-PAGE and Western Blot analysis

Protein expression on bacterial cultures and in all purification, steps was assessed by SDS-PAGE. Samples were loaded into 15% acrylamide gels. PageRuler™ Unstained Protein Ladder (26614) and Prestained Protein Ladder (26616) were used as markers to monitor protein separation and estimate protein size. The separation was performed at 200 V for 45 min using Mini-PROTEAN® 3 cell system Biorad; Cressier, Switzerland). For western blot analysis, the proteins were subsequently transferred on a 0.45 µm nitrocellulose membrane. The wet transfer was performed at 30 V overnight in a cold room, using Mini Trans-Blot® cell system (Biorad; Cressier, Switzerland). A fluorescently labeled goat anti-mouse secondary antibody (Li-cor IRDye® 680RD goat anti-mouse IgG secondary antibody) was used to detect either the His-tag using a mouse anti-His antibody (Qiagen, #34660) as a primary antibody or the Myc-tag using a mouse anti-Myc antibody (Invitrogen, #132500). The membranes were then analyzed at a

wavelength of 700 nm by the Odyssey® CLx Imaging System (LI-COR Biotechnology – GmbH, Bad Homburg, Germany).

S5. Nanobody Expression and Purification

S5.1. Protein expression

A preculture was prepared from pipette tip punctured glycerol stock in 10 ml of LB supplemented with the appropriate antibiotic. This bacterial culture was grown overnight at 37°C under vigorous shaking (150 rpm). The following day, 900 ml of TB containing 0.4% glycerol and supplemented with the appropriate antibiotic was inoculated with 1 % (v/v) of the preculture and the OD_{600} was monitored during growth by removing aliquots aseptically until it reached a value of 1-1.3 (late-log phase) (3-4 h at 37°C)[1-4]. Since the expression of the nanobodies was under the control of the T7 promoter, isopropyl β-d-1-thiogalactopyranoside (IPTG) was used for the induction of protein expression by adding 1 mM IPTG to the culture after decreasing the incubator temperature from 37°C to 25°C. The expression was carried out for 24 hours. After protein expression, the bacterial suspension was split into 2 x 450 ml and cell cultures were harvested by centrifugation at 5000 g for 20 min, at 4°C (ThermoFisher, Germany). The bacterial pellet was then recovered, weighed, and stored at -20°C until further processing. Note that the volumes used, as well as IPTG concentration and incubation temperature, were used as a routine scale-up. These conditions can be modified or adapted according to the desired purposes.

S5.2. Protein purification

Bacterial lysis was carried out by mechanical disruption using the French Press. First, the thawed pellet was resuspended in 30 ml lysis buffer consisting of 96% (v/v) buffer A (20 mM Tris (base), 500 mM NaCl, pH 8.0), 4% (v/v) buffer B (20 mM Tris (base), 500 mM NaCl, 500 mM imidazole, pH 8.0), a point of spatula of DNase and 1 ml of 100 mM PMSF. The cells were then passed twice through the French Press (Thermo, Milford, USA) and the resulting lysate was centrifuged at 12500 g for 30 min at 4°C (ThermoFisher, Germany) to separate the cell debris from the soluble protein fraction. The supernatant was then filtrated through a 0.45 µm syringe filter unit and passed through a 5 ml His-tag affinity column (HisTrap

™FF 5 mL) (Cytiva, USA) pre-equilibrated with 4% buffer B (buffer A containing 20 mM imidazole), to isolate the protein by immobilized metal ion affinity chromatography (IMAC) (Ni-affinity chromatography). The purification was performed on an ÄKTA Fast Protein Liquid Chromatography (FPLC) (GE Healthcare, Uppsala, Sweden) using a linear gradient of 15 column volume (CV) of imidazole starting from 4% buffer B to 100 % buffer B, at a flow rate of 2 ml/min. Non-specifically bound impurities were removed prior to elution by washing the column with more than 10 CVs of buffer A containing 20 mM imidazole. After elution, the fractions containing the protein of interest were collected, pooled, and dialyzed overnight at 4°C against 1 l of Buffer E (25 mM Hepes, 133 mM NaCl, pH 7.4) with 2 changes over 24 h, in order to remove the imidazole (3.5 kDa molecular weight cut-off (MWCO) membrane). The protein was then concentrated by ultrafiltration (10kDa MWCO, Amicon® Ultra-15) quantified by NanoDrop® One^{C} (Thermo Scientific, USA), snap-frozen in small aliquots, and stored at -80°C. At all purification steps, samples were taken, analyzed by SDS-PAGE, and stained by Coomassie blue staining.

S5.2.1. M7D12H_{WT} Nanobody purification

A very clear and sharp peak was observed on the chromatogram starting from fraction 14 to 24 (***Figure S1a***), showing a high level of protein expression by the bacteria (BL21(DE3)$_{WT}$). At ~37% imidazole concentration, the M7D12H_{WT} nanobody started to elute (fractions 14 to 24) and clear and highly pure protein at around 18 kDa was observed on the gel when these fractions were analyzed by SDS-PAGE (***Figure S1b***) (see red boxes). All these results suggested that the M7D12H_{WT} nanobody was successfully expressed by *E.coli* and efficiently purified from the IMAC column in a single step. A MALDI-TOF analysis (section 6.1), and a western blot (section 6.2) were performed to confirm the integrity of the expressed recombinant nanobody.

(a)

(b)

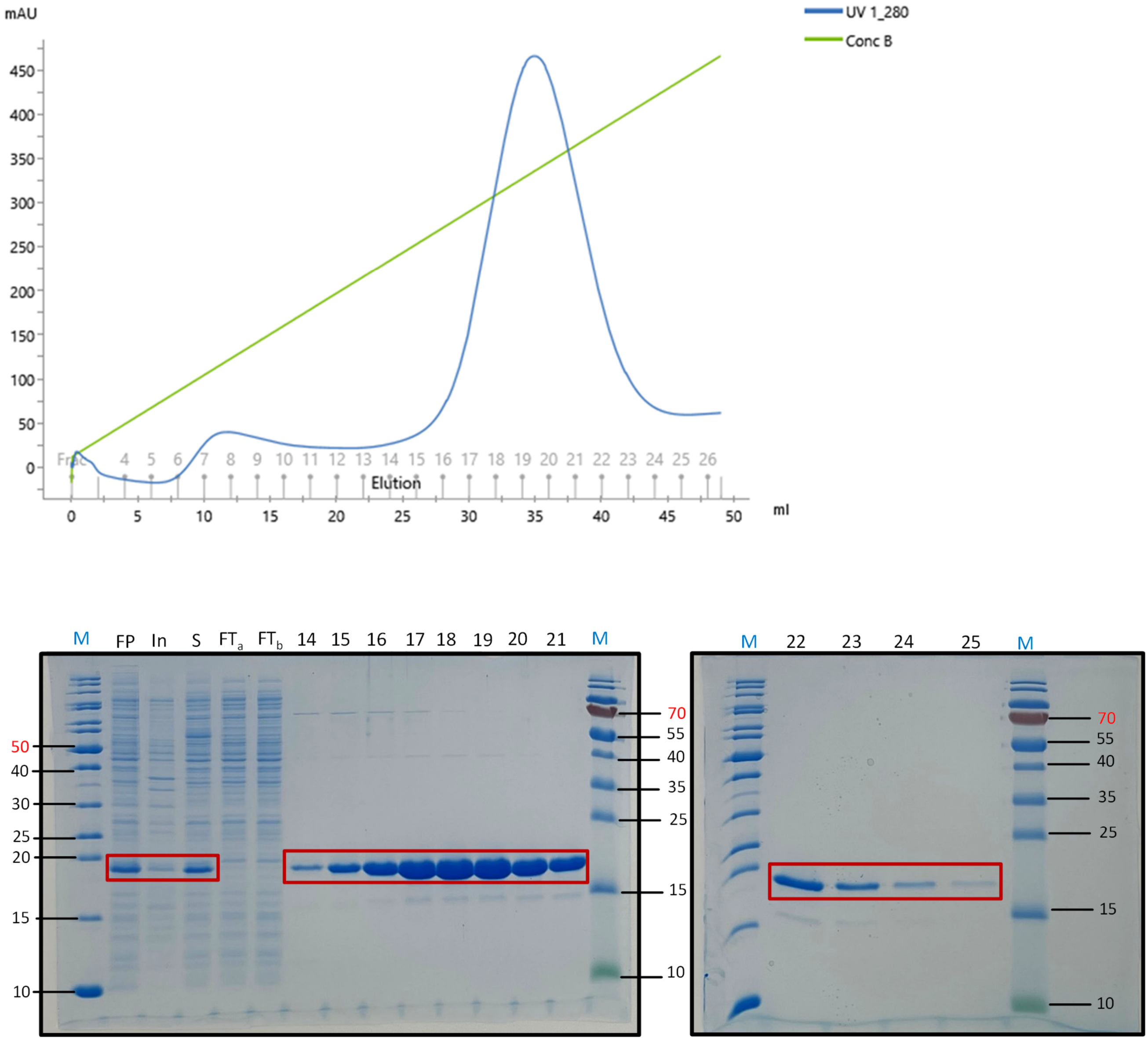

***Figure S1: (a)** Nickel column chromatogram of **M7D12H** wild-type nanobody. **(b)** SDS-PAGE (15%) gel of the purification. M: Low range marker; FP: total extract after the French press; In: pellet; S: filtered supernatant; FTa: Flow through; FTb: Flow through collected after a second pass of the FTa through the column; 14-25: collected fractions.*

S5.2.2. Mono-substituted variants purification

The expression and purification of the mutants were performed in the same manner as for the $M7D12H_{WT}$ nanobody to meet the iontophoretic study conditions of the wild-type nanobody and to avoid any variations due to the expression and purification protocols.

(a)

(b)

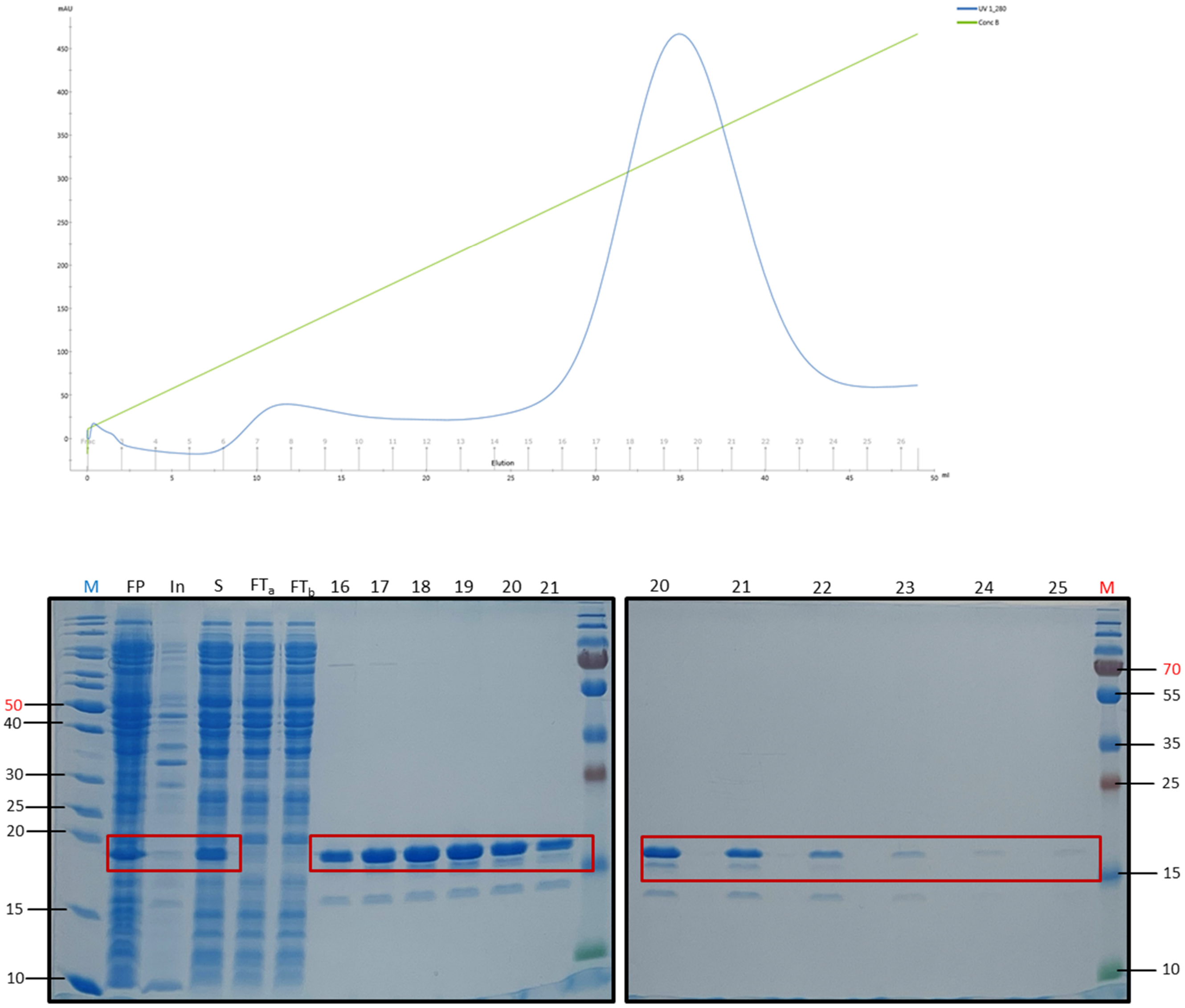

*Figure S2: (a) Nickel column chromatogram of the **R54E mutant**. (b) SDS-PAGE gel (15%) of protein purification. M: 26614 and 26619 PageRuler Ladder; FP: total extract after the French press; In pellet; S: supernatant; FTa: Flow-through; FTb: Flow-through collected after a second pass of the FTa through the column; 16-25: collected fractions.*

(a)

(b)

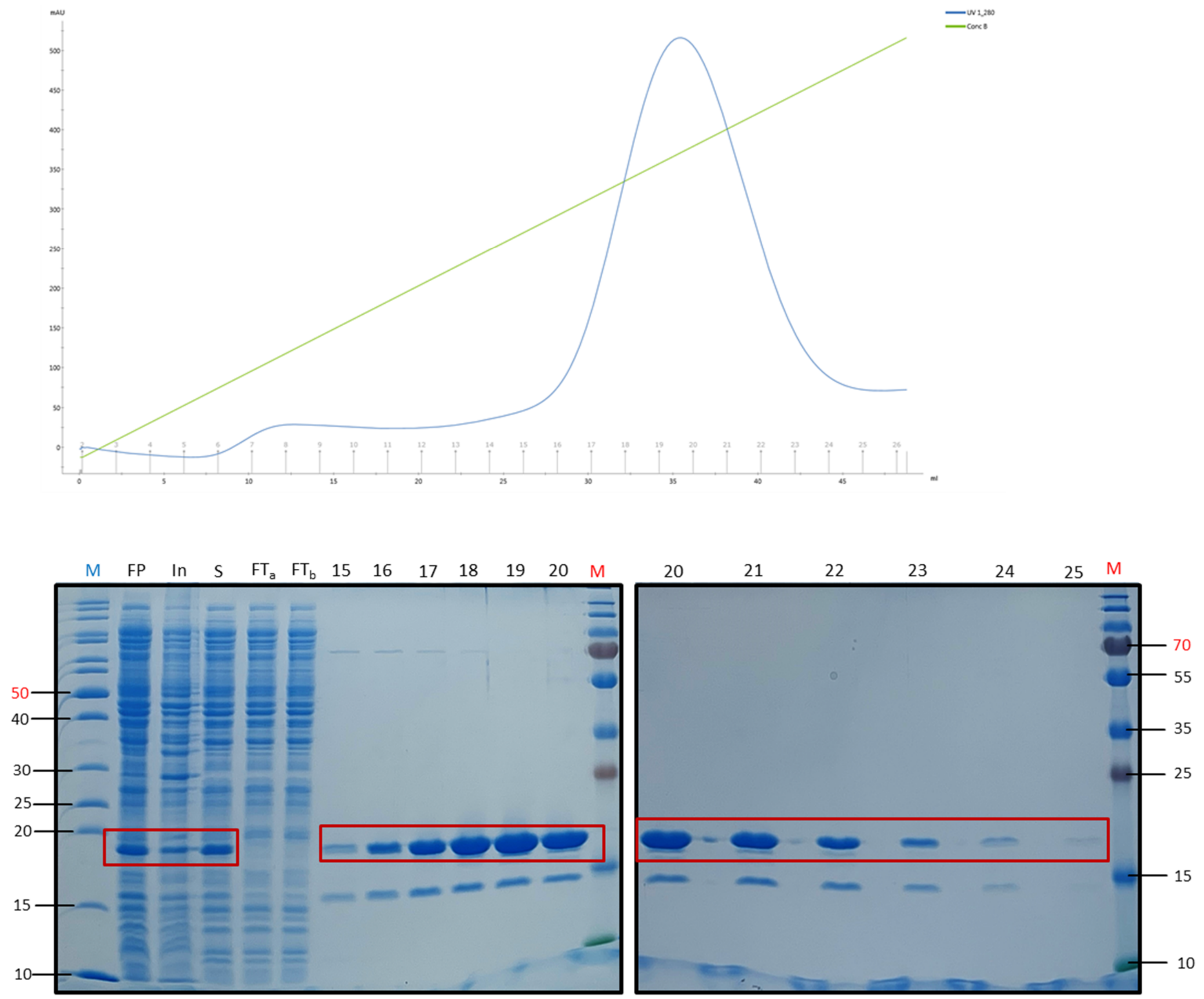

***Figure S3: (a)** Nickel column chromatogram of the **K65E mutant**. **(b)** SDS-PAGE gel (15%) of protein purification. M: 26614 and 26619 PageRuler Ladder; FP: total extract after the French press; In pellet; S: supernatant; FTa: Flow-through; FTb: Flow-through collected after a second pass of the FTa through the column; 15-25: collected fractions.*

(a)

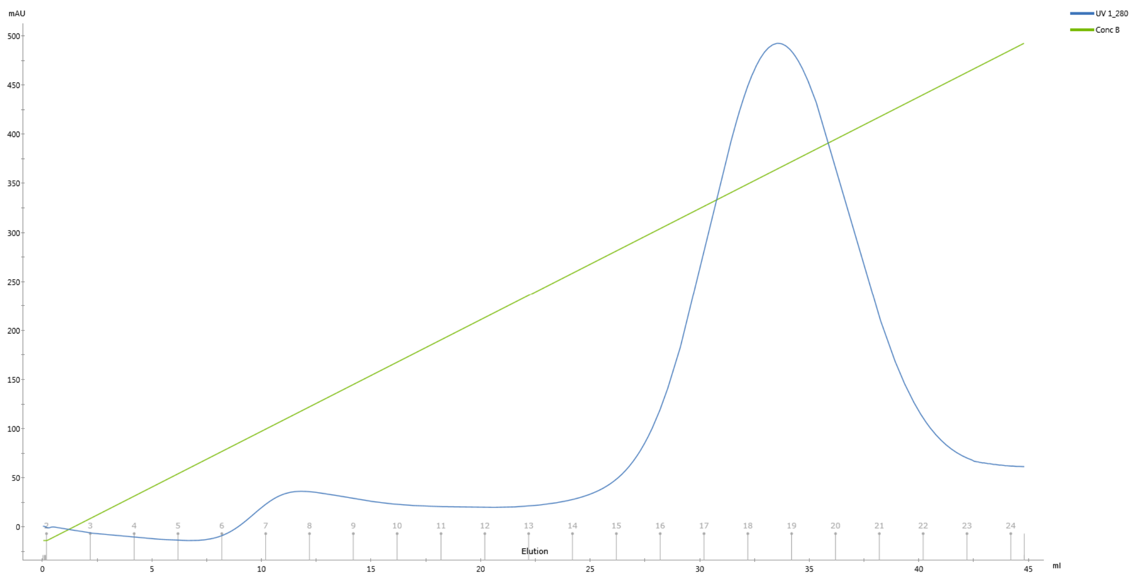

(b)

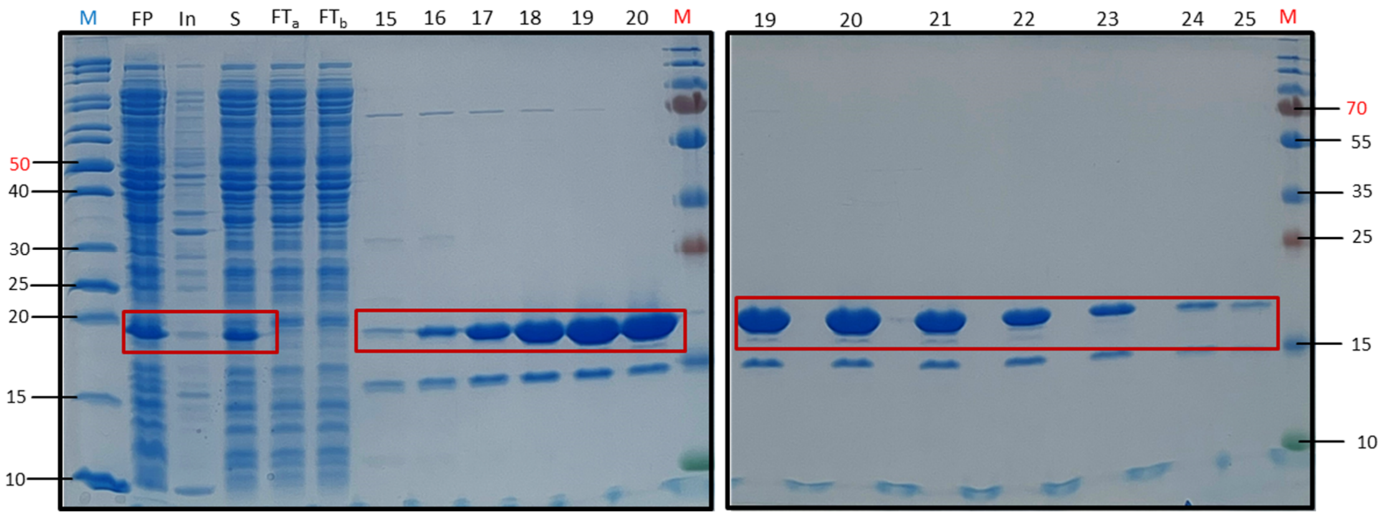

***Figure S4: (a)** Nickel column chromatogram of the **S102E mutant**. **(b)** SDS-PAGE gel (15%) of protein purification. M: 26614 and 26619 PageRuler Ladder; FP: total extract after the French press; In pellet; S: supernatant; FTa: Flow-through; FTb: Flow-through collected after a second pass of the FTa through the column; 15-25: collected fractions.*

As for wild-type M7D12H, clear and sharp peaks were obtained on the chromatograms starting from fractions 16 to 25 for R54E (***Figure S2a***), fractions 15 to 24 for K65E (***Figure S3a***), and fractions 15 to 23 for S102E (***Figure S4a***), pointing to a high level of protein expression by the wild-type BL21(DE3). Similar to the M7D12H$_{WT}$, the mono-substituted variants started to elute at ~ 37% imidazole concentration

(starting from fraction 15), and clear and relatively pure proteins at around 18 kDa were identified on the gel (***Figure S2b, S3b,*** and ***S4b***)(see red boxes). All these results suggested that these mutants were successfully expressed by *E.coli* and purified in a single step. A western blot (section 6.2), and ESI-LC-MSMS analysis (section 6.3) were performed to confirm the integrity of the expressed recombinant nanobodies.

S6. Nanobody characterization

S6.1. MALDI-TOF analysis of M7D12H$_{WT}$ nanobody

To confirm the integrity and identity of the expressed and purified protein, samples were sent to the Mass Spectrometry core facility (Faculty of Sciences, University of Geneva) for mass analysis (MALDI-TOF; Bruker Autoflex) according to their standard protocols. The analysis (**Figure S5**) revealed a protein molecular weight of 17485.0 Da which was in excellent agreement with the predicted value of 17487.06 Da. This result indicated that intact protein was expressed without any structural modifications.

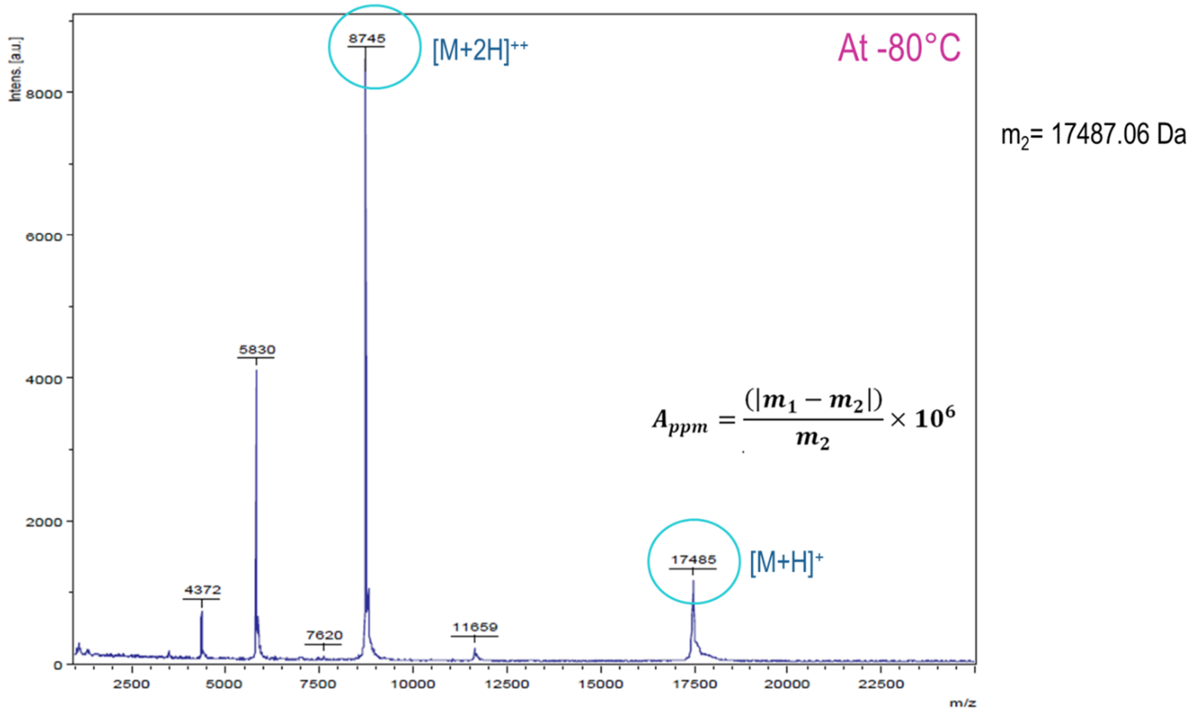

Figure S5: *Characterization of purified M7D12H$_{WT}$ nanobody stored at -80°C performed by MALDI-TOF analysis (MALDI-TOF-MS spectrum).*

S6.2. Confirmation of M7D12H$_{WT}$ nanobody Expression and its Variants

The identity of the recombinant proteins was confirmed by western blot, using a mouse anti-MycTag antibody (Invitrogen, #132500) followed by Li-cor IRDye® 680RD goat anti-mouse IgG detection. As shown in the figure below (**Figure S6**), proteins with an approximate molecular weight of 18 kDa were detected on the membrane, thus confirming the successful expression of the nanobodies.

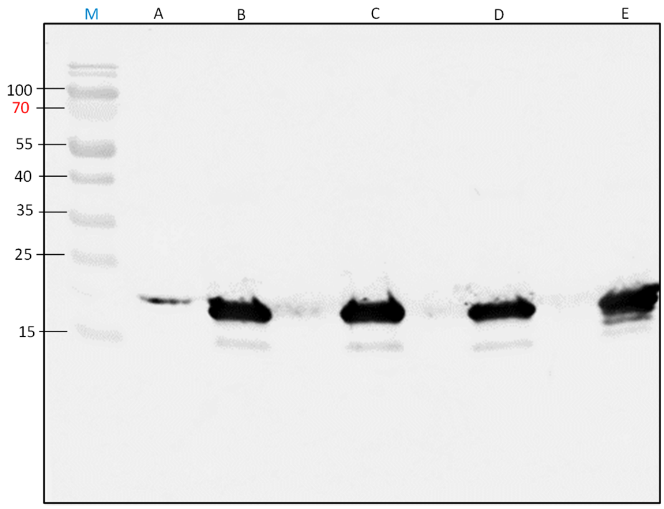

Figure S6: *Western blot analysis of the expression of M7D12$_{WT}$ and its variants, using mouse anti-Myc antibody.* ***M:*** *PageRuler™ prestained protein ladder 26616;* ***(A)*** *PBST* ***(B)*** *M7D12H$_{WT}$;* ***(C)*** *S102E;* ***(D)*** *R54E;* ***(E)*** *K65E.*

S6.3. ESI-LC-MS/MS analysis of the nanobodies

To confirm the presence of the recombinant proteins (i.e., M7D12H$_{WT}$ and the mutants), the Coomassie-stained gel bands were subjected to ESI-LC-MSMS analysis at the Proteomics Core Facility (Faculty of Medicine, University of Geneva). The gel bands were cut, and in-gel digestion was performed using trypsin or chymotrypsin. Subsequently, the peptides were analyzed by nanoLC-MSMS using an easynLC1000 (Thermo Fisher Scientific) coupled with a Q-Exactive HF mass spectrometer (Thermo Fisher Scientific).

For database searches, Mascot (Matrix Science) was utilized, employing the *Escherichia Coli* database as well as the sequences of the expressed proteins. Data were analyzed and validated with Scaffold (Proteome Software) with 1% of protein FDR and at least 2 unique peptides per protein with 0.1% of peptide FDR. Mass spectrometry on the excised bands successfully identified the wild-type protein M7D12H, as well as the variants K65E and R54E in their corresponding bands and with peptides containing their respective specific amino acids. According to the Proteomics Core Facility platform, the S102E mutant *"**was also identified in its band, and the set of detected peptide sequences excluded the presence of the R54E and K65E mutants in this sample. However, as expected, the set of detected***

peptides was common to both the wild-type M7D12H protein and the S102E mutant and thus it was not possible to distinguish between these two proteins”, even after chymotrypsin digestion.

Nevertheless, considering the molecular weights the difference between the wild-type protein and the S102E mutant (MW_{S102E} =17528.9 Da - MW_{M7D12H} =17486.9 Da = **42 Da**) corresponded exactly to the difference between a glutamic acid residue and a serine (MW_E = 147.1 Da – MW_S = 105.1 Da = **42 Da**). This finding led to the conclusion that the S102E variant was indeed expressed.

S7. Validation of the ELISA method

S7.1. Specificity and matrix effect

(a)

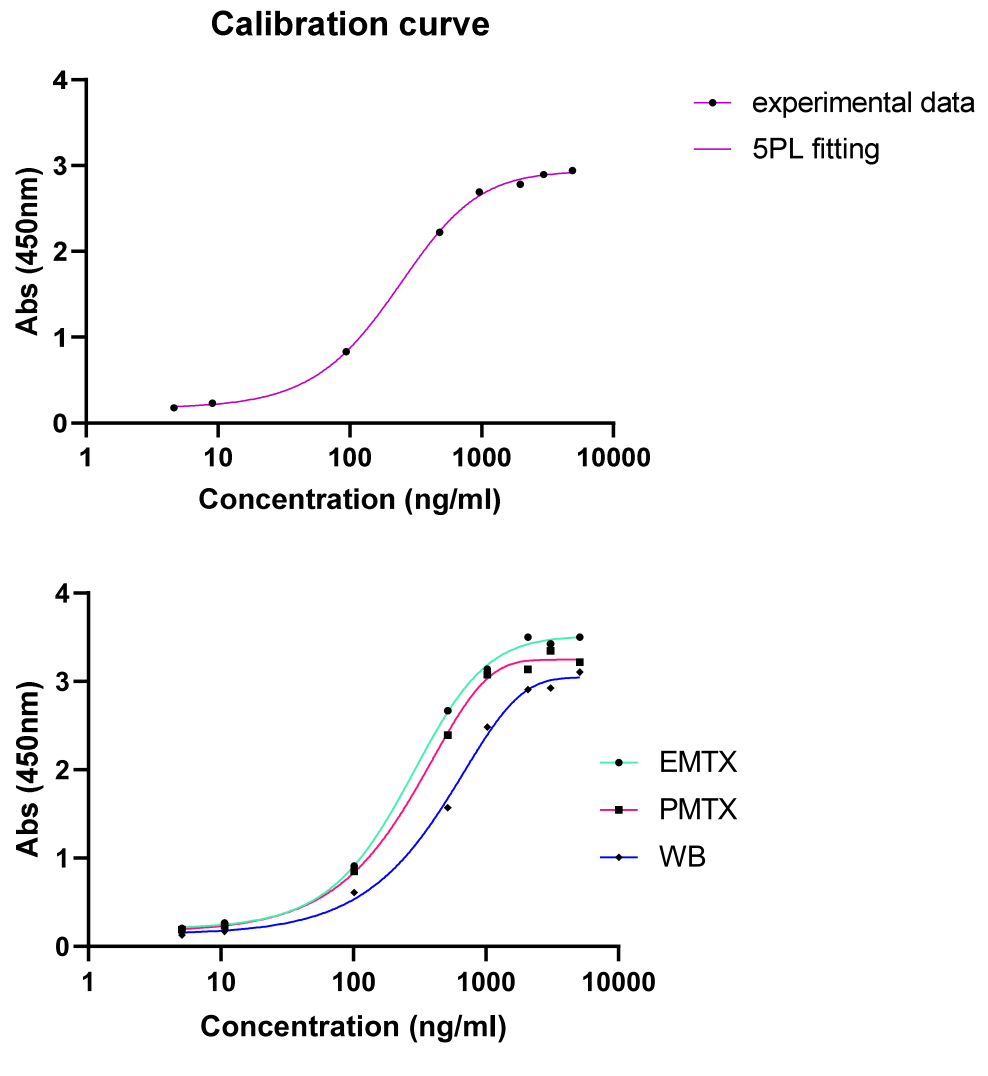

(b)

***Figure S7: (a)** Typical calibration curve obtained experimentally and fitted by the 5PL model. **(b)** Standard calibration curve observed in wash buffer (PBST), skin extraction matrix (EMTX), and receiver permeation medium (PMTX).*

Figure S7a shows a standard calibration curve constructed using a concentration range of 5 – 5000 ng/ml of M7D12H$_{WT}$ nanobody. The regression analysis was performed using the five-parameter logistic (5PL) function, described by **Equation S1**. The nanobody concentration was calculated using **Equation S2**, with

"x" representing the protein concentration and *"f(x)"* the corresponding signal (Abs). Skin interference was clearly observed when carrying out the calibration curves in the different media (***Figure S7b***), demonstrating a matrix effect. This is likely due to non-covalent interactions between skin proteins and the analyte, which impede the interaction of the nanobody with immunoassay antibodies. It was then decided to prepare different calibration curves in their respective matrices in each experiment, to take into account this effect.

Equation S1:

$$f(x) = d + \frac{(a-b)}{\left(1+\left(\frac{x}{c}\right)^{b}\right)^{e}}$$

Equation S2:

$$x = \mathrm{c} \times \left(\left(\frac{a-d}{y-d}\right)^{\frac{1}{e}} - 1\right)^{\frac{1}{b}}$$

With:

d: maximal signal

a: minimal signal

c: the concentration (x) at the inflection point

b: the slope of the hill

e: asymmetry parameter

S7.2. Accuracy and precision

The intra-plate (within-run) and inter-plate (between-run) precision and accuracy in each matrix were measured and the data are presented in **Table S6** below.

a)

	Intra-plate (WB)			Inter-plate (WB)		
$[V_HH]_{theo}$ (ng/ml)	$[V_HH]_{meas}$ (mean ± SD)	RSD (%)	RECOVERY (%)	$[V_HH]_{meas}$ (mean ± SD)	RSD (%)	RECOVERY (%)
**5**	4.86 ± 0.43	8.9	94.0	5.06 ± 0.68	13.5	97.8
**100**	112.24 ± 5.96	5.3	107.7	115.01 ± 3.89	3.4	110.3
**1000**	1115.46 ± 44.95	4.0	110.3	1068.68 ± 83.91	7.9	105.7

b)

	Intra-plate (EMTX)			Inter-plate (EMTX)		
$[V_HH]_{theo}$ (ng/ml)	$[V_HH]_{meas}$ (mean ± SD)	RSD (%)	RECOVERY (%)	$[V_HH]_{meas}$ (mean ± SD)	RSD (%)	RECOVERY (%)
**5**	5.62 ± 0.33	5.9	109.2	4.96 ± 0.58	11.6	96.5
**100**	103.65 ± 2.13	2.1	101.5	107.14 ± 3.65	3.4	104.9
**1000**	1011.72 ± 14.74	1.5	98.9	1056.85 ± 40.52	3.8	103.3

c)

	Intra-plate (EMTX)			Inter-plate (EMTX)		
$[V_HH]_{theo}$ (ng/ml)	$[V_HH]_{meas}$ (mean ± SD)	RSD (%)	RECOVERY (%)	$[V_HH]_{meas}$ (mean ± SD)	RSD (%)	RECOVERY (%)
**5**	5.56 ± 0.33	5.9	109.6	4.91 ± 0.57	11.7	96.7
**100**	97.32 ± 4.30	4.4	95.9	103.57 ± 5.95	5.7	102.1
**1000**	1022.25 ± 16.91	1.7	100.1	1055.56 ± 96.54	9.1	103.4

Table S6: *Intra and inter-plate accuracy and precision for protein quantitation in* ***a)*** *wash buffer (WB);* ***b)*** *extraction matrix (EMTX);* ***c)*** *permeation matrix (PMTX)* with ELISA.

The results obtained were within the limits of acceptance according to ICH guidelines M10 on the bioanalytical method; therefore, the ELISA method was considered accurate and precise.

S8. Protein stability in the presence of skin and current

A control gel of M7D12H_{WT} and the different variants was used as the reference to investigat the effect of exposure to skin and current application *(**Figure S8a**)*. Exposure to porcine skin for M7D12H_{WT} nanobody and R54E had a slightly deleterious effect after 8 hours *(**Figure S8b**)*. Indeed, small fragments are visible on the gel at t=8h for these two proteins compared to t=0. However, the profile of the gel is the same as that of the control gel, which means that the skin did not affect the integrity of the proteins but perhaps the temperature: the latter increased up to 35°C during the experiment, which would probably have affected the proteins. Application of a current at 0.5 mA/cm^2 for 8 hours had no detrimental effect on the integrity of the wild-type protein or the three mutants *(**Figure S8c**)*.

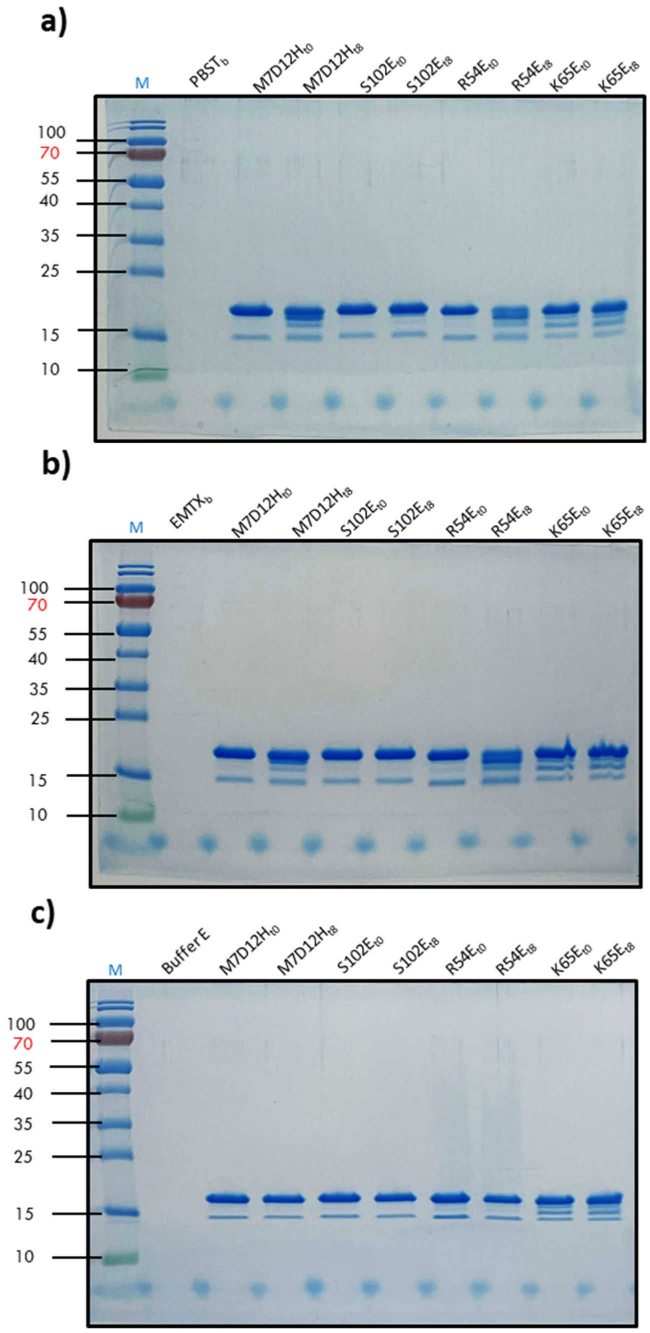

Figure S8: *SDS-PAGE analysis (15%, 5% stack) and Coomassie blue staining of M7D12H wild-type protein and its variants in the presence of the skin and current;* ***a)*** *Control;* ***b)*** *Proteins in the presence of skin* ***c)*** *Proteins in the presence of current.* ***PBST$_b$:*** *PBST blank;* ***EMTX$_b$****: Skin extraction blank;* ***Buffer E****: Buffer blank (25 mM Hepes, 133 mM NaCl, pH7,4);* ***$_{t0}$****: Sample collected at t=0h;* ***$_{t8}$****: Sample collected at t=8h.*

S9. References

1. Gutierrez-Gonzalez M, Farias C, Tello S, Perez-Etcheverry D, Romero A, Zuniga R, et al. Optimization of culture conditions for the expression of three different insoluble proteins in Escherichia coli. *Sci Rep*. **2019**;*9*:16850.
2. Francis DM, Page R. Strategies to optimize protein expression in E. coli. *Curr Protoc Protein Sci*. **2010**;*Chapter 5*:5241-5.24.29.
3. San-Miguel T, Pérez-Bermúdez P, Gavidia I. Production of soluble eukaryotic recombinant proteins in E. coli is favoured in early log-phase cultures induced at low temperature. *Springerplus*. **2013**;*2*:89.
4. Galloway CA, Sowden MP, Smith HC. Increasing the yield of soluble recombinant protein expressed in E. coli by induction during late log phase. *Biotechniques*. **2003**;*34*:524-6, 8, 30.
